# Electroluminescence of ordered ZnO nanorod array/p-GaN light-emitting diodes with graphene current spreading layer

**DOI:** 10.1186/1556-276X-9-630

**Published:** 2014-11-25

**Authors:** Jing-Jing Dong, Hui-Ying Hao, Jie Xing, Zhen-Jun Fan, Zi-Li Zhang

**Affiliations:** 1 School of Science, China University of Geosciences (Beijing), 29 Xue Yuan Road, Haidian District, Beijing, 100083, China

**Keywords:** Zinc oxide, Nanorod array, Light-emitting diodes, Graphene

## Abstract

Ordered ZnO nanorod array/p-GaN heterojunction light-emitting diodes (LEDs) have been fabricated by introducing graphene as the current spreading layer, which exhibit improved electroluminescence performance by comparison to the LED using a conventional structure (indium-tin-oxide as the current spreading layer). In addition, by adjusting the diameter of ZnO nanorod array in use, the light emission of the ZnO nanorod array/p-GaN heterojunction LEDs was enhanced further. This work has great potential applications in solid-state lighting, high performance optoelectronic devices, and so on.

**PACS:**

78.60.Fi; 85.60.Jb; 78.67.Lt; 81.10.Dn

## Background

ZnO is regarded as one of the most promising semiconductor material for the application of ultraviolet light-emitting diodes (LEDs) due to its excellent optical and electrical properties, including a direct wide bandgap (3.37 eV) and a high exciton binding energy (60 meV) at room temperature. Especially ordered ZnO nanorod arrays, which have a lower defect density, an improvement in the light extraction efficiency of the LEDs, and a broadband suppression in reflection, are the most attractive [1-5]. In addition, by choosing an optimal diameter of the ZnO nanorod arrays, electroluminescence (EL) emission from the LED device can be enhanced further, because the waveguiding property of ZnO nanorods is closely related to the diameter [5,6]. Recently, ZnO nanorod array-based LEDs have been reported by many groups, which presents improved EL performance compared with the ZnO film-based LEDs [5-7]. However, the fabrication of ZnO nanorod array-based LEDs with a traditional indium-tin-oxide (ITO) current spreading layer is very complicated. Since ZnO nanorods do not form a continuous layer, it is necessary to protect the nanorods and isolate electrical contacts on the ZnO nanorods from the underlying substrate. This can be achieved by spin coating an insulating polymer layer to fill up the gaps between nanorods. And then, an oxygen plasma etching was used to remove the insulating polymer coated on the surface of ZnO nanorods and expose the nanorod tips for contact formation. However, the complete filling and moderate etching of the insulating polymer are very difficult to control, and moreover, the plasma may introduce damage of ZnO nanorods leading to the degradation of the electrical properties of LEDs [6,8]. Recently, graphene has emerged as a promising material for transparent conductive electrode, considering its excellent characteristics such as high transparency and low sheet resistance [9-11]. For ZnO nanorod array-based LEDs, the device fabrication process can be considerably simplified by adopting graphene as a current spreading layer instead of ITO, since the spin coating and etching of insulating polymer can be completely omitted.

In this letter, the highly ordered and vertical aligned ZnO nanorod arrays were first grown on p-GaN substrates via a facile hydrothermal process assisted by the inverted self-assembled monolayer (SAM) template. Then, ordered ZnO nanorod array/p-GaN heterojunction LEDs were fabricated by introducing few-layer graphene as a current spreading layer, and improved EL performance was achieved compared with that of the ZnO nanorod array-based LED with a conventional structure. Finally, the performance of the ZnO nanorod array/p-GaN LEDs was optimized by adjusting the diameter of the ZnO nanorod array in use. This work provides a route for developing high performance optoelectronic devices based on ZnO nanorods.

## Methods

Highly ordered ZnO nanorod arrays on the p-GaN substrates were fabricated by a hydrothermal process using the TiO_2_ ring template deriving from the polystyrene (PS) microsphere SAM as reported in our previous report [12]. Herein, the diameters of ZnO nanorods were tuned by varying the solution concentration during hydrothermal growth and reactive ion etching (RIE) of PS microspheres, which has been described in detail in our previous reports [6,12]. To grow the ZnO nanorod arrays, the aqueous solutions of Zn(NO_3_)_2_ · 6H_2_O (99.5% purity, Aldrich, St. Louis, MO, USA) and hexamethylenetetramine (HMTA) (99.5% purity, Aldrich) with the identical concentration were used.

The fabrication of ZnO nanorod array-based LEDs with graphene current spreading layer was as follows. Graphene was synthesized on Cu foil by chemical vapor deposition (CVD) in a tubular quartz reactor, using methane as a carbon source in the H_2_ and Ar atmosphere. To transfer grapheme from Cu foil to the top of ZnO nanorod array, the grapheme samples were firstly spin coated with polymethylmethacrylate (PMMA) on the surface. After completely etching the Cu foils by dipping in the FeCl_3_ solution, the graphene films were transferred onto the ZnO nanorod arrays. Finally, the PMMA layers were dissolved in acetone leaving graphene on the ZnO nanorod array. To improve the electrical contact, Au/Ti contacts were thermally evaporated onto the graphene.

### Characterization

Surface morphologies of the samples were characterized by scanning electron microscopy (SEM, Hitachi FE-S4800, Hitachi, Ltd., Chiyoda, Tokyo, Japan). The EL measurements of ZnO-based LEDs were carried out at room temperature (RT) using a Hitachi F4500 fluorescence spectrophotometer. The photoluminescence (PL) spectra were acquired by exciting with a 325-nm He-Cd laser with a power of 30 mW at RT.

## Results and discussion

**Figure 1:**
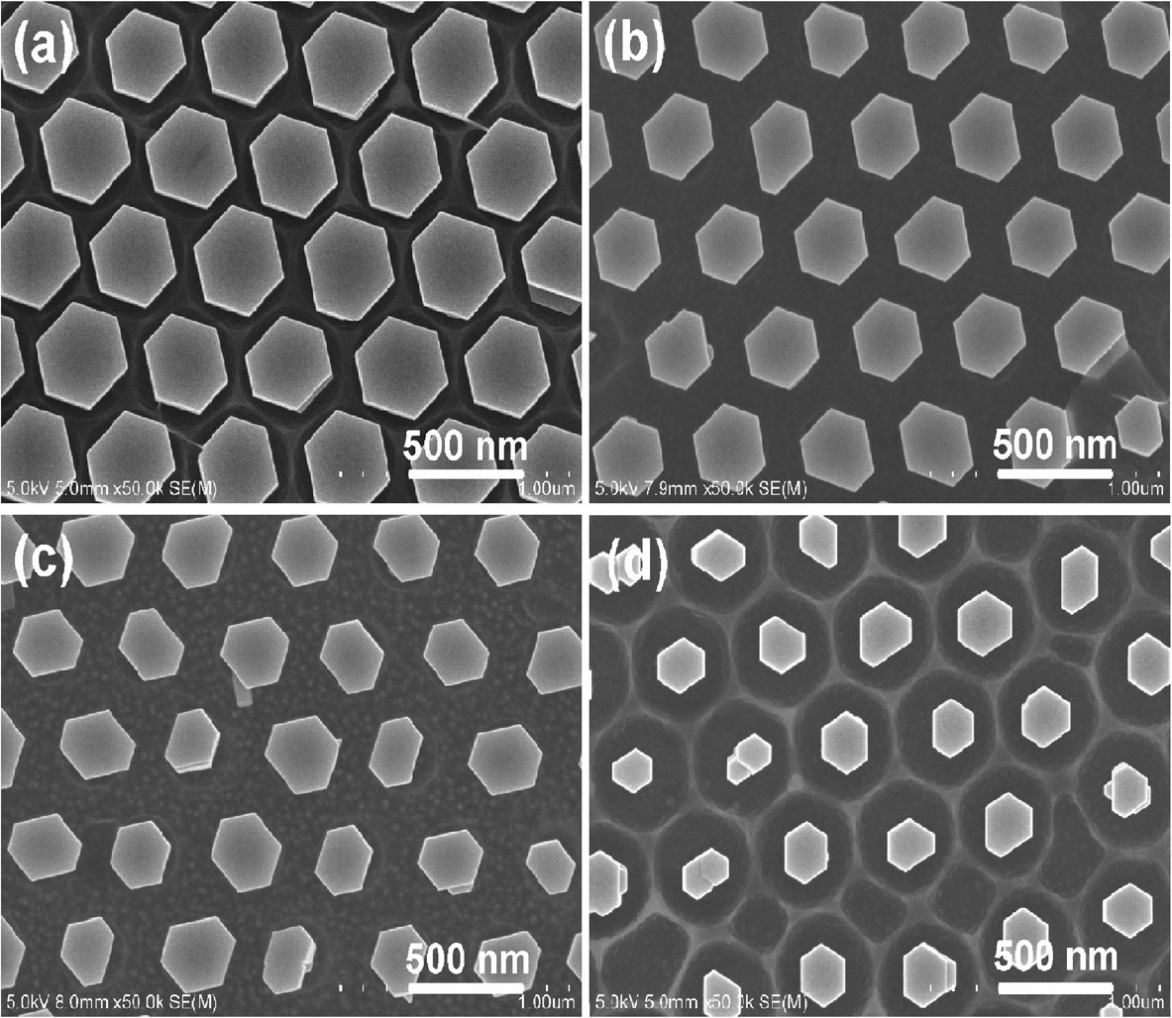
**Ordered and aligned ZnO nanorod arrays on the p-GaN substrates grown at 50°C for 6 h.** With the reactant concentration of **(a)** 0.05 M and **(b)** 0.035 M by using 500 nm PS microspheres without RIE; **(c)** 0.05 M and **(d)** 0.035 M by RIE of 500 nm PS microspheres for 1.5 min.

**Figure 2:**
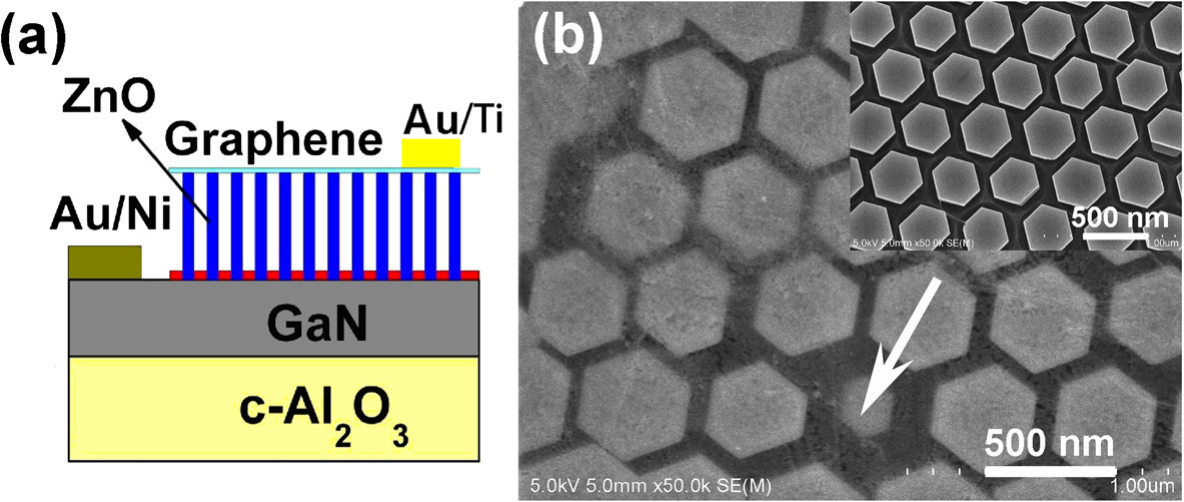
**Schematic structure of the LEDs and SEM image of the ZnO nanorod array. (a)** Schematic structure of the ZnO nanorod array/p-GaN LEDs with graphene as the current spreading layer. **(b)** Top view SEM image of the ordered ZnO nanorods with and without (inset) graphene.

EL emission of the ZnO nanorod array-based LED can be enhanced further by choosing an optimal diameter of the ZnO nanorods, as stated earlier. It has been demonstrated in our previous work that the diameter of ZnO nanorods can be tuned by either varying the solution concentration during hydrothermal growth or RIE of PS microspheres [6,12]. As shown in Figure 1, ordered and aligned ZnO nanorod arrays on the p-GaN substrates were grown at 50°C for 6 h, and the diameter was reduced from 380 nm (Figure 1a) to 300 nm (Figure 1b) by varying the solution concentration from 0.05 to 0.035 M, and 220 nm (Figure 1c) by RIE of PS microspheres for 1.5 min. Here, the diameter of the PS microspheres in use is 500 nm. Taking advantage of both two measures mentioned above, ZnO nanorod array with a diameter of 170 nm was obtained, as shown in Figure 1d. It can be seen that all the ZnO nanorod arrays reserve hexagonal periodicity and evenly distribution inheriting from the PS microsphere SAM, and the spacing between two neighboring nanorods is 500 nm which is predefined by the diameter of the PS microspheres. All of the ZnO nanorod arrays are hexagonal faceted perfectly aligned normal to the underlying substrate, indicating that each ZnO nanorod is a single crystal of wurtzite ZnO with growth direction along [0001]. Besides, the hexagonal nanorods are well oriented with their side faces parallel to each other, demonstrating the perfect epitaxial growth of ZnO nanorods on the GaN substrate, which has been confirmed by the X-ray diffraction (XRD) measurements in our previous report [12].The highly ordered ZnO nanorod arrays on the p-GaN substrate were then used to construct LED devices with graphene as the current spreading layer, and the schematic structure of the device is illustrated in Figure 2a. To confirm the complete unfolding of the graphene layer on the top of ZnO nanorod array, a typical SEM image of ZnO nanorod array covered with graphene is shown in Figure 2b, and the corresponding SEM image without graphene is given in the inset of Figure 2b for comparison. It can be seen that both of the ZnO nanorod arrays are hexagonal faceted with a smooth surface and a clear edge, indicating a tight contact between graphene and ZnO nanorods. In addition, for the small defects of the ZnO nanorod array, as indicated by the arrows in Figure 2b, the graphene layer plays an important role in modifying the gap. Obviously, a continuous graphene film was covered on the top of separated ZnO nanorods and plays a role of current spreading as an interconnecting material of ZnO nanorods, so under forward current injection, each ZnO nanorod acts as a light emitter.

**Figure 3:**
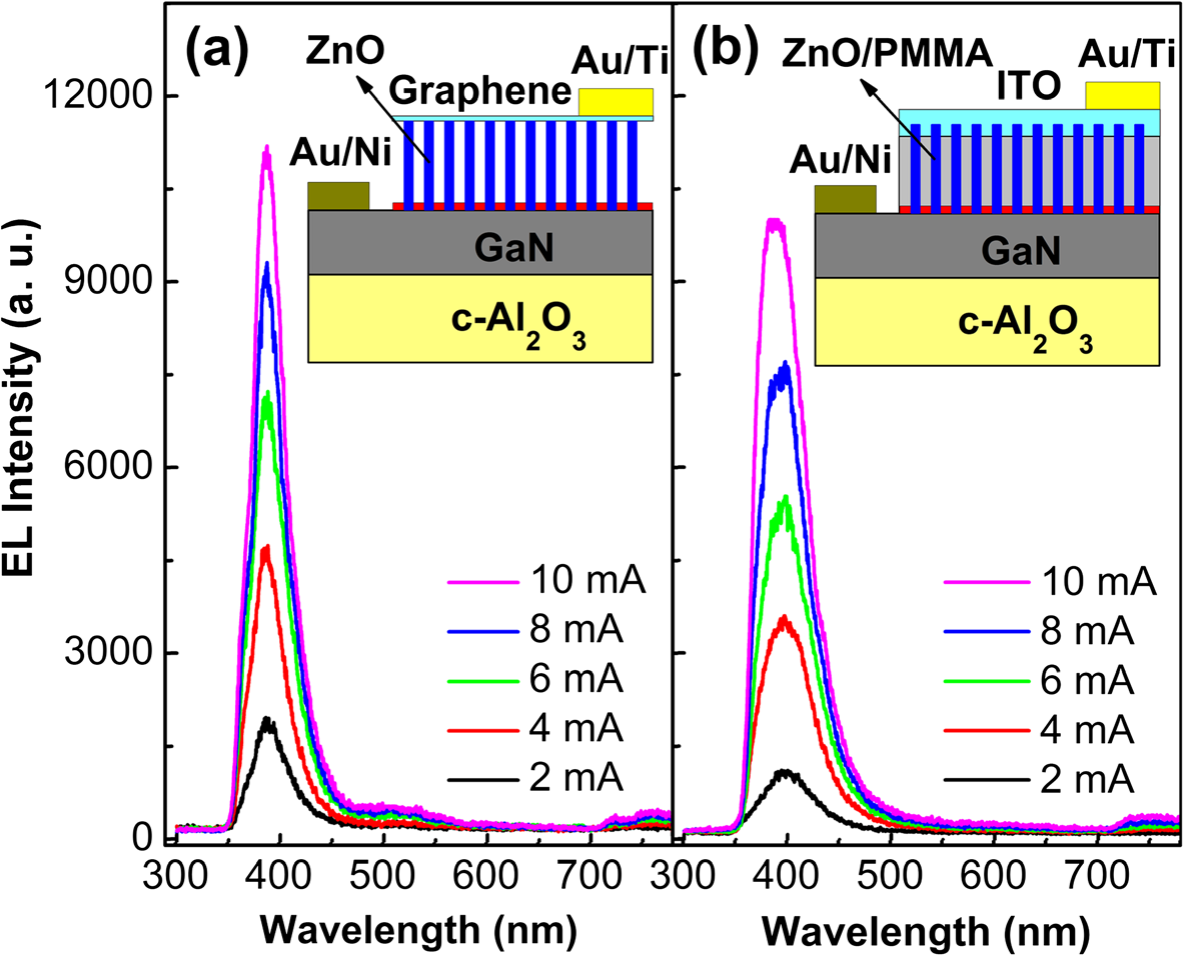
**EL spectra of the ZnO nanorod array/p-GaN LEDs.** With **(a)** graphene as the current spreading layer and **(b)** traditional structure, under various currents ranging from 2 to 10 mA. The insets show the schematic structures of the corresponding LEDs, respectively.

The EL spectra of ordered ZnO nanorod array/p-GaN LED (the diameter of the ZnO nanorods in use is 300 nm) with graphene current spreading layer under various currents ranging from 2 to 10 mA are shown in Figure 3a. It can be seen that these spectra exhibit a distinct UV emission peak centered at about 390 nm, which has been observed frequently from the EL spectra of ZnO-based LEDs and can be attributed to the near-band edge (NBE) emission from ZnO [13-15]. Besides, the strong EL emission even at low currents can be attributed to the low density of the interfacial defects, the improved carrier injection efficiency through the nanosized junctions [16,17], and the increase in the light extraction efficiency by virtue of the waveguiding property of ZnO nanorods [6,15]. For comparison, a reference LED with a traditional structure (containing a PMMA insulating layer and a top ITO current spreading layer) was fabricated, and the corresponding EL spectra and its schematic structure are presented in Figure 3b. Obviously, the EL emission from the ZnO nanorod array-based LED with graphene current spreading layer is stronger than that of the traditional structure at all injection currents, which can be due to the stable, reliable, and low-resistance ohmic contacts between graphene and ZnO nanorods, as well as the higher transmittance of graphene than ITO especially in the UV region [8].

**Figure 4:**
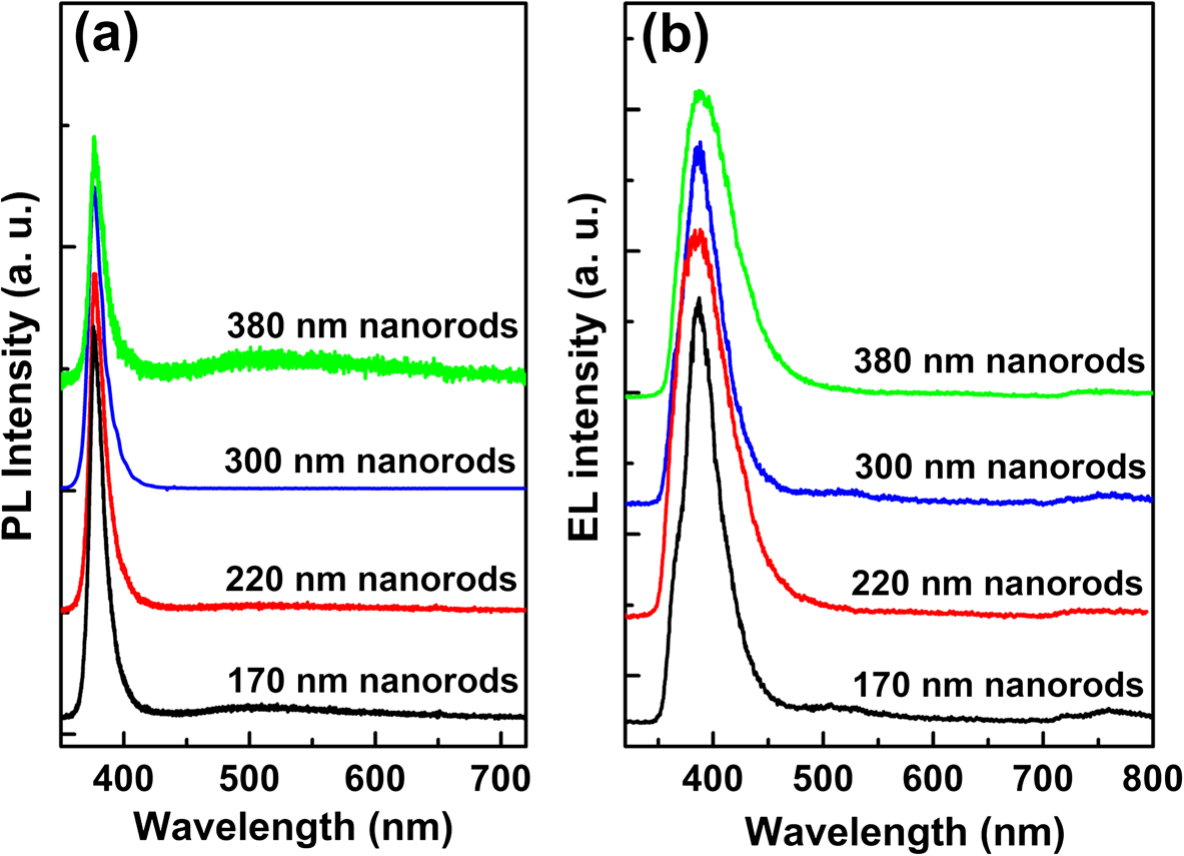
**PL spectra of the ZnO nanorod arrays and EL spectra of the corresponding LEDs. (a)** PL spectra of the ZnO nanorod arrays on the p-GaN substrate with different diameters. **(b)** EL spectra of the LEDs based on ZnO nanorod arrays with different diameters at the injection current of 6 mA.

As mentioned earlier, one of the advantages of the ordered ZnO nanorod array over the ZnO film is the enhanced light emission by virtue of the waveguiding effect of nanorods. Besides, by choosing an optimal diameter of the ZnO nanorod arrays, EL emission can be enhanced further, because the waveguiding property of ZnO nanorods is closely related to the diameter [5,18]. So, the optical properties of the ZnO nanorod arrays with different diameters have been studied, and the corresponding PL and EL spectra are presented in Figure 4. As can be seen in Figure 4a, the PL spectra of the ZnO nanorod arrays on GaN substrates with the diameters of 170, 220, 300, and 380 nm present a unique UV emission at 380 nm, which is due to the NBE emission of ZnO. The absence of any other peaks from ZnO, for example, the defect-related visible emission within the experimental resolution, indicates the high crystal quality of the ZnO nanorod arrays on p-GaN substrates. Figure 4b presents the EL spectra of the ZnO nanorod array-based LEDs with different diameters (170, 220, 300, and 380 nm) at the injection current of 6 mA, and all the four diodes show a dominant NBE emission at 390 nm. Compared with the PL spectra, there is a 10-nm red shift of the EL NBE emission, which is caused by the junction-heating effect under a constant injection current [14,19] and difference between PL and EL processes (the PL process depends on the recombination of nonequilibrium carriers in the surface layer, whereas the EL process is determined via the carrier recombination within the space charge region of heterojunction [20]).

The most important feature, however, of the results presented in Figure 4 is that the intensity of the EL and PL NBE emission vary with the diameter of the ZnO nanorod arrays. As known, the thin film-based heterojunction LEDs are suffering from the low-light extraction efficiency as limited by the total internal reflection, so the nanorod/thin film heterostructures are proposed to be a promising method to enhance the light emission considering the feasible waveguiding properties of ZnO nanorods [5]. Under the single-mode waveguide cavity conditions [18], according to the following formula:

(1) $$V=\pi \frac{a}{\lambda_{0}}{\left(n_{1}^{2}-n_{2}^{2}\right)}^{\frac{1}{2}}=2.405$$

86.5% of the light would be confined within a 170 nm ZnO nanorod, where *V* is the single mode cut-off value, *a* is the diameter of the nanowire, *λ*_0_ is the free space wavelength of the propagating light (here, we set it 390 nm), and *n*_1_ (2. 0) and *n*_2_ (1.0) are the effective refractive index of the ZnO nanorod and air, respectively. Consistent with the theoretical calculation above, Figure 4 presents that the ZnO nanorod array with the diameter of 170 nm shows the strongest PL and EL emission. So, 170 nm is the optimal diameter of the ZnO nanorod array for the application of heterojunction LEDs.

## Conclusions

In conclusion, we fabricated highly ordered ZnO nanorod arrays with controllable diameter on p-GaN substrates via a facile hydrothermal process assisted by the inverted SAM template. On this basis, ZnO nanorod array/p-GaN heterojunction LEDs were fabricated by introducing graphene as the current spreading layer, which exhibit improved EL performance compared with the LED with a conventional structure. In addition, by choosing an optimal diameter of 170 nm, light emission of the ZnO nanorod array/p-GaN heterojunction LEDs was enhanced further. This work has great potential applications in solid-state lighting, high performance optoelectronic devices, and so on.

## Abbreviations

CVD: Chemical vapor deposition; EL: Electroluminescence; LEDs: Light-emitting diodes; ITO: Indium-tin-oxide; NBE: Near-band edge; PL: Photoluminescence; PMMA: Polymethylmethacrylate; PS: Polystyrene; RIE: Reactive ion etching; RT: Room temperature; SEM: Scanning electron microscopy; SAM: Self-assembled monolayer; UV: Ultraviolet; XRD: X-ray diffraction.

## Competing interests

The authors declare that they have no competing interests.

## Authors' contributions

J-JD designed the experiment, carried out the experiment, analyzed the results, and participated in the draft of the manuscript; H-YH supervised the research and revised the manuscript; JX, Z-LZ, and Z-JF offered the technique supports. All authors read and approved the final manuscript.
